# Treatment of acne keloidalis nuchae and dissecting cellulitis of the scalp with diclofenac sodium gel: a case series

**DOI:** 10.1016/j.jdcr.2023.09.008

**Published:** 2023-09-27

**Authors:** Eduardo A. Michelen-Gómez, Zelma C. Chiesa Fuxench, Karina J. Cancel-Artau, Amara Guerrero, Omer Ibrahim, Luis E. Santaliz-Ruiz, Francisco Colon-Fontanez

**Affiliations:** a Department of Dermatology, School of Medicine, University of Puerto Rico, San Juan, Puerto Rico; b Department of Dermatology, University of Pennsylvania Perelman School of Medicine, Philadelphia, Pennsylvania; c Universidad Central del Caribe, School of Medicine, San Juan, Puerto Rico; d Department of Dermatology, Rush University Medical Center, Chicago, Illinois

**Keywords:** Acne keloidalis nuchae, dissecting cellulitis of the scalp, cicatricial alopecia, diclofenac

## Introduction

Acne keloidalis nuchae (AKN) and dissecting cellulitis of the scalp (DCS) are chronic inflammatory skin diseases that share a similar etiology. DCS is more common in adult Black men but can affect women and patients of other races or ethnicities.^1^, ^2^, ^3^ It is associated with neutrophilic occlusion of the pilosebaceous unit and follicular hyperkeratosis. DCS is divided into an early stage consisting of follicular plugging and suppurative follicular/perifollicular abscesses and a second stage, characterized by granulation tissue, scarring, sinus-tract formation, and progression to cicatricial alopecia.^3^ AKN often presents as papules and pustules of varying sizes that coalesce to form firm, keloid-like plaques on the occipital scalp and posterior aspect of the neck of men with curly hair.^4^ Patients often describe the lesions of AKN as painful and pruritic, and when extensive areas are involved, may result in patches of alopecia or, in severe cases, complete hair loss. It is associated with perifollicular lymphocytic and plasmocytic infiltration**.** The management of DCS and AKN remains a challenge because therapeutic alternatives are few and data are limited concerning efficacy and tolerability.^3^^,^^4^

Diclofenac sodium gel is a topical, nonsteroidal anti-inflammatory drug indicated for the treatment of osteoarthritic joint pain.^5^ Given its anti-inflammatory properties, it may be a useful treatment option in patients with DCS or AKN who have failed or are not candidates for conventional therapy. Here, we report our experience with 3 patients with DCS and 1 patient with AKN treated with topical diclofenac sodium gel monotherapy during a 3-month course.

### Patient 1

**Fig 1:**
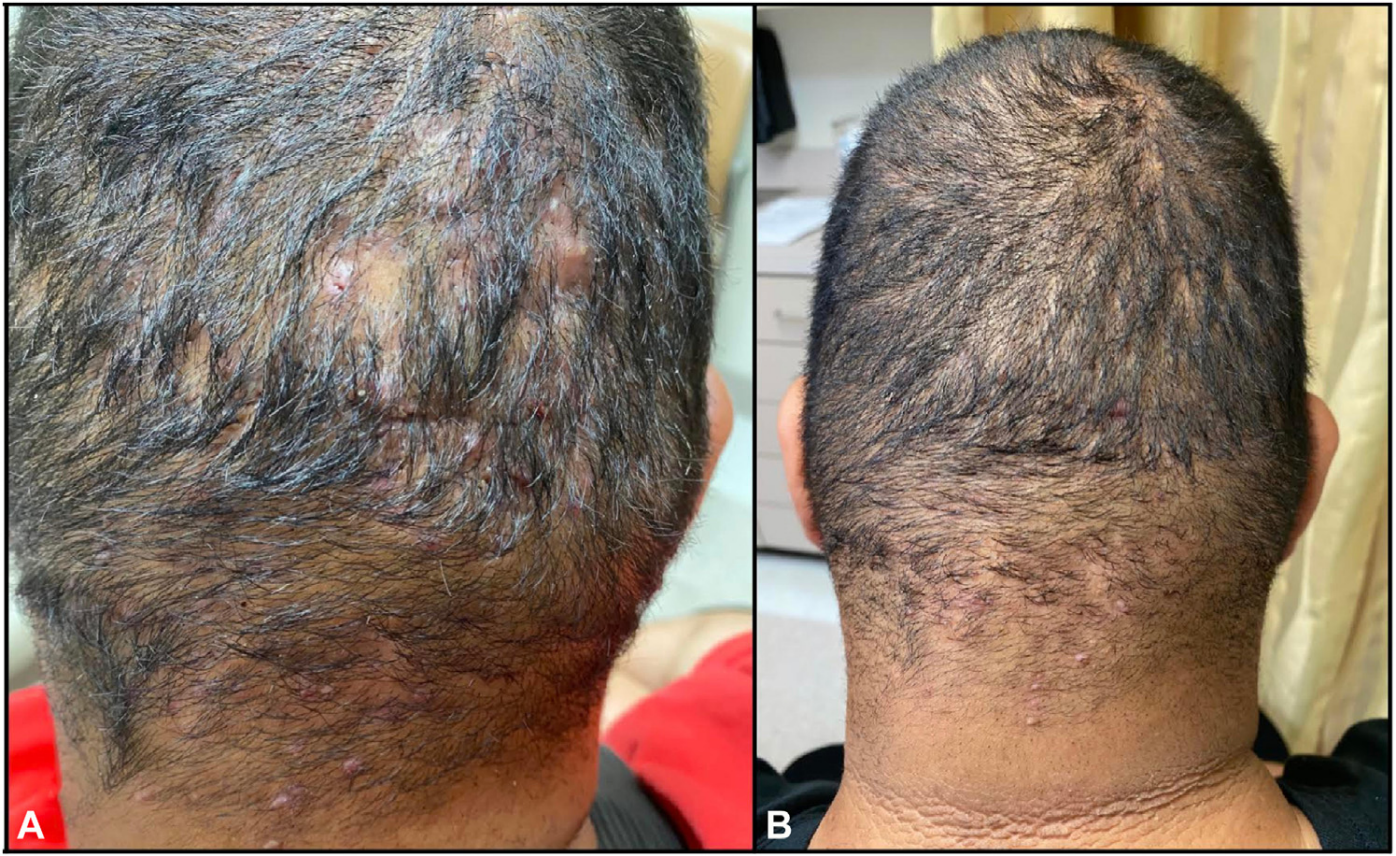
Patient with a history of multiple, tender, erythematous papules, pustules, and subcutaneous fluctuant nodules with patches of hair loss. **A,** Patient from case 1 prior to treatment. **B,** Patient from case 1 after a 3-month course with diclofenac gel.

A 24-year-old man presented to the dermatology clinic with a 5-month history of painful “bumps” on the scalp. Physical examination revealed multiple, tender, erythematous papules, pustules, and subcutaneous fluctuant nodules with patches of alopecia localized to the occipital scalp and posterior aspect of the neck (Fig 1, *A*). The patient reported pain as 5 of 10 on a numerical rating scale (NRS). He was previously treated with a 4-month course of doxycycline 100 mg once daily with a partial response. Based on clinical findings, DCS was diagnosed. After a thorough discussion, he consented to twice-a-day monotherapy treatment with 1% diclofenac sodium gel for 3 months. At 1-month follow-up, he reported improvement in his condition and denied any treatment-related side effects besides mild burning sensation on application to draining lesions. Few, tender, erythematous papules, and pustules were noted on examination, and pain was rated as 3 of 10 NRS. At 3-month follow-up, tenderness had resolved, the number of papules had decreased, and hair growth was evident (Fig 1, *B*). Based on clinical improvement, the patient was recommended to decrease the application of diclofenac gel to once a day, every other day as maintenance therapy. After 4 months of maintenance therapy, treatment effectiveness was sustained.

### Patient 2

A 17-year-old man with a 1-year history of DCS presented to our clinic with painful lesions and abscesses on the posterior scalp and hair loss. Prior treatment for DCS included isotretinoin 40 mg daily, 1% clindamycin solution daily, and 4% benzoyl peroxide wash daily; however, he reported noncompliance because of concern for isotretinoin-related side effects. Physical examination of the occipital scalp revealed multiple erythematous papules, pustules, alopecia patches, and scarring areas. The pain was graded as 2 of 10 NRS. After discussing treatment options, he proceeded with monotherapy treatment with twice-a-day application of 1% diclofenac sodium gel for 3 months. At 3-month follow-up, symptoms had improved and increased hair growth was observed (Table I). No treatment-related side effects were reported with application of diclofenac gel. Physical examination revealed scattered, nontender, erythematous papules, pustules, and an increase in hair density over patches of alopecia on the occipital scalp. The application of diclofenac gel was decreased to once a day on alternate days as maintenance therapy. After 3 months of ongoing maintenance therapy, therapeutic success was maintained.

**Table I:** Reported pain on a numerical rating scale before and after diclofenac gel monotherapy

Patient	Reported pain at initial visit	Reported pain at 3-mo follow-up visit
Patient #1	5/10	3/10
Patient #2	2/10	No pain
Patient #3	6/10	2/10
Patient #4	4/10	1/10

### Patient 3

**Fig 2:**
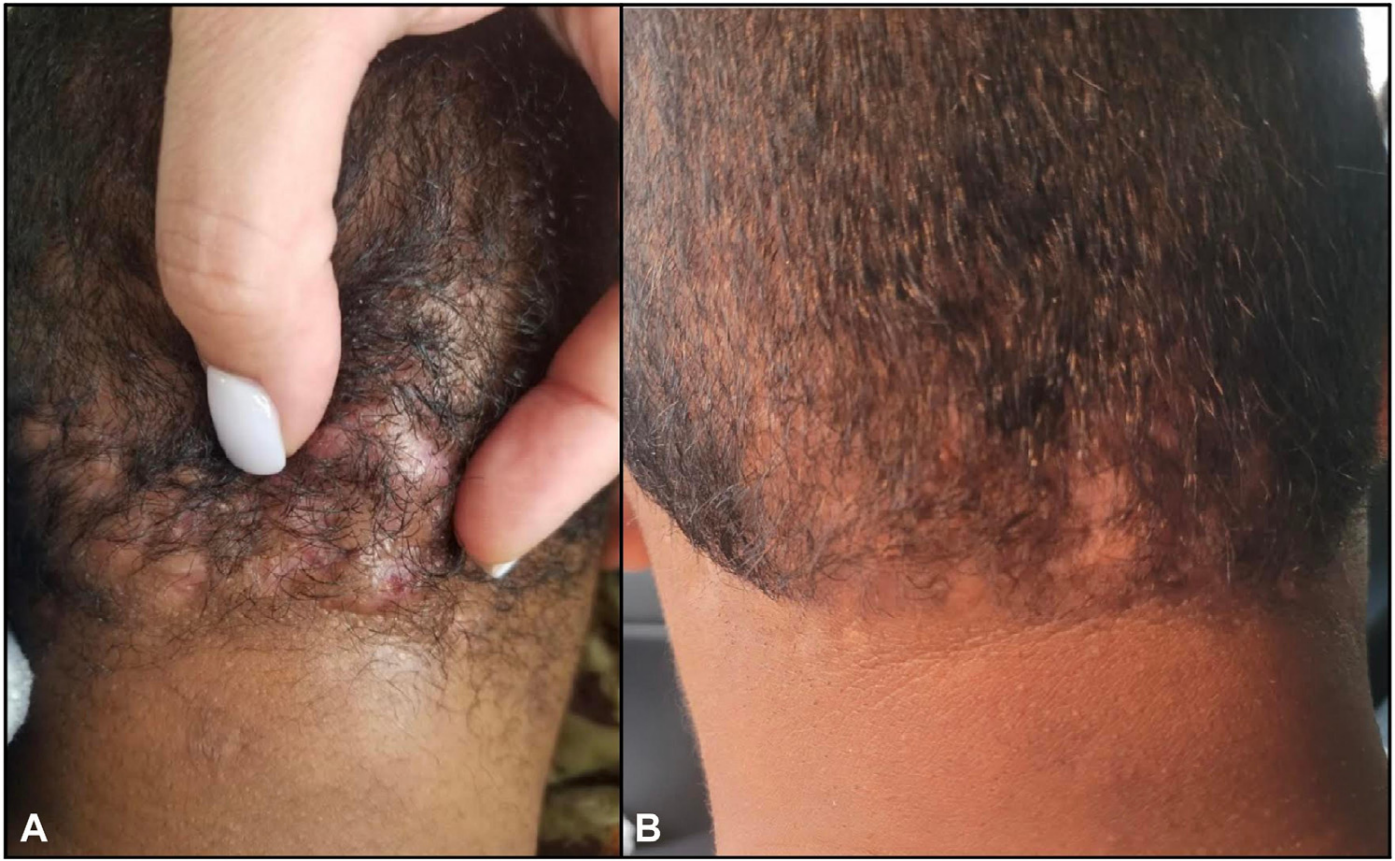
An 18-year-old man with a 2-year history of DCS. **A,** Patient prior to treatment. **B,** Patient after a 3-month course with diclofenac gel.

An 18-year-old man with a 2-year history of DCS presented to our clinic for evaluation. Prior treatments included minocycline 100 mg daily, isotretinoin 60 mg daily, and intralesional triamcinolone, all with partial success. Because of markedly increased triglyceride levels, isotretinoin was discontinued, and, subsequently, he developed recurrence of painful, tender, boggy nodules on the occipital scalp with areas of scarring and hair loss (Fig 2, *A*). Pain was rated as 6 of 10 NRS. After discussing treatment options, he consented to twice-a-day application of diclofenac sodium gel 1% monotherapy. At 3-month follow-up, the patient reported improvement in his condition and denied any treatment-related adverse effects (Table I). Physical examination was remarkable for 1 pustule on the occipital scalp with areas of alopecia showing hair growth (Fig 2, *B*). The patient continued once daily application of diclofenac gel on alternate days as maintenance therapy for 3 months with excellent response and minimal recurrence.

### Patient 4

**Fig 3:**
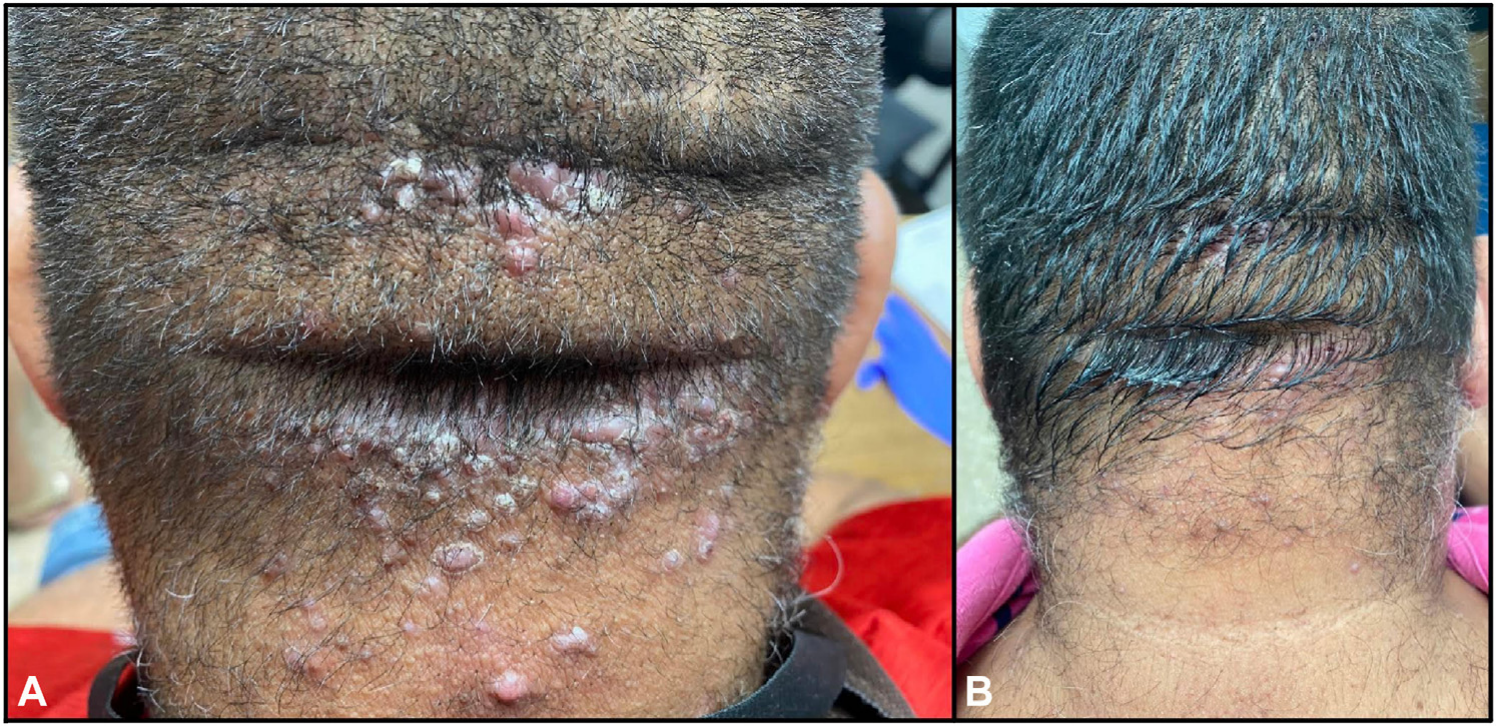
A 52-year-old man with a history of multiple tender, dome-shaped, keratotic, follicular-based papules. **A,** Pretreatment photograph. **B,** Three-month progress with diclofenac gel.

A 52-year-old man with diabetes and hypertension presented for evaluation of an 8-month history of pruritic, painful small “bumps” on the scalp and neck. Physical examination revealed scattered, tender, dome-shaped, keratotic, follicular-based papules, coalescing into plaques, on the occipital scalp and posterior aspect of the neck (Fig 3, *A*). No areas of alopecia were noted. Pain was reported as 4 of 10 NRS. Based on clinical findings, AKN was diagnosed. In addition to limiting mechanical trauma to the area, monotherapy treatment with 1% diclofenac sodium gel was recommended. At the 1-month follow-up, the patient reported using diclofenac once daily, and noting symptom improvement, no new lesions, and no treatment-related side effects (Table I). Physical examination was remarkable for fewer nontender, erythematous, papules on the occipital scalp and posterior aspect of the neck. The examination remained unchanged at 3-month follow-up (Fig 3, *B*). The patient was continued on maintenance with a once a day, every other day, application of diclofenac gel with a sustained response after 5 months of ongoing maintenance therapy.

## Discussion

To the best of our knowledge, the use of diclofenac gel has not been previously reported in patients with DCS and AKN. Current therapeutic alternatives for DCS include isotretinoin, tumor necrosis factor- alpha (TNF-a) inhibitors, intralesional corticosteroids, oral antibiotics, and oral high-dose zinc sulfate. Their effectiveness is based on a few, limited case reports with high-recurrence rates on discontinuation of therapy.^6^^,^^7^ Surgery, radiation, and photodynamic therapy have also been used in refractory cases.^6^ In a similar manner, current treatment options for AKN are also associated with high failure and relapse rates and mostly include oral retinoids, tetracycline antibiotics, and high-potency topical or intralesional corticosteroids.^4^^,^^8^, ^9^, ^10^

Although the exact mechanism is not fully understood, diclofenac is thought to inhibit the enzyme cyclooxygenase, disrupting the arachidonic acid cascade.^11^ Consequently, thromboxanes, prostaglandins, and prostacyclins formation is decreased, producing an anti-inflammatory effect. Diclofenac also inhibits the aggregation and activation of neutrophils, inhibits chemotaxis, alters lymphocyte activity, and decreases proinflammatory cytokine levels, leading to analgesic and antipyretic properties.^5^^,^^11^^,^^12^ We hypothesized that these diclofenac-related effects might result in an overall decrease in the neutrophilic and mixed-lymphocytic infiltrate in hair follicles of patients with DCS and AKN, leading to the observed remission in our patients.

All our patients with DCS showed substantial improvement with diclofenac gel monotherapy, with most lesions resolving within a 3-month treatment period. A similar response was observed in our patient with AKN after 1-month of therapy. Similar clinical responses have been observed with systemic agents, such as isotretinoin and TNF-a inhibitors, in DCS and AKN.^13^^,^^14^ Nevertheless, because diclofenac gel is topically applied, side effects related to steroid-induced skin atrophy and systemic absorption are negligible**.** It may also be a more affordable option for patients with none-to-limited insurance coverage.

Finally, we would like to emphasize that all 4 patients were considered to have mild forms of the disease and therefore we have no evidence of diclofenac’s potential efficacy in treating severe or more refractory cases nor did we examine the recurrence rate after therapy discontinuation. Given these promising results, efforts are underway to develop a larger placebo-controlled clinical trial to evaluate the efficacy and safety of diclofenac gel in this population and in patients with mild-to-moderate hidradenitis suppurativa.

## Conflicts of interest

None.

## References

[1] Chicarilli Z.N. (1987). Follicular occlusion triad: hidradenitis suppurativa, acne conglobata, and dissecting cellulitis of the scalp. Ann Plast Surg.

[2] Gamissans M., Romaní J., López-Llunell C., Riera-Martí N., Sin M. (2022). Dissecting cellulitis of the scalp: a review on clinical characteristics and management options in a series of 14 patients. Dermatol Ther.

[3] Badaoui A., Reygagne P., Cavelier-Balloy B. (2016). Dissecting cellulitis of the scalp: a retrospective study of 51 patients and review of literature. Br J Dermatol.

[4] Ogunbiyi A. (2016). Acne keloidalis nuchae: prevalence, impact, and management challenges. Clin Cosmet Investig Dermatol.

[5] Tieppo F.V., Davani S., Towery C., Brown T.L. (2017). Oral versus topical diclofenac sodium in the treatment of osteoarthritis. J Pain Palliat Care Pharmacother.

[6] Scheinfeld N. (2014). Dissecting cellulitis (perifolliculitis capitis abscedens et suffodiens): a comprehensive review focusing on new treatments and findings of the last decade with commentary comparing the therapies and causes of dissecting cellulitis to hidradenitis suppurativa. Dermatol Online J.

[7] Thomas J., Aguh C. (2019). Approach to treatment of refractory dissecting cellulitis of the scalp: a systematic review. J Dermatolog Treat.

[8] Callender V.D., Young C.M., Haverstock C.L., Carroll C.L., Feldman S.R. (2005). An open label study of clobetasol propionate 0.05% and betamethasone valerate 0.12% foams in treatment of mild to moderate acne keloidalis. Cutis.

[9] Alexis A., Heath C.R., Halder R.M. (2014). Folliculitis keloidalis nuchae and psuedofolliculitis barbae: are prevention and effective treatment within reach?. Dermatol Clin.

[10] Goh M.S.Y., Magee J., Chong A.H. (2005). Keratosis follicularis spinulosa decalvans and acne keloidalis nuchae. Australas J Dermatol.

[11] Kienzler J.L., Gold M., Nollevaux F. (2010). Systemic bioavailability of topical diclofenac sodium gel 1% versus oral diclofenac sodium in healthy volunteers. J Clin Pharmacol.

[12] Zacher J., Altman R., Bellamy N. (2008). Topical diclofenac and its role in pain and inflammation: an evidence-based review. Curr Med Res Opin.

[13] Alsantali A., Almalki B., Alharbi A. (2021). Recalcitrant dissecting cellulitis of the scalp treated successfully with adalimumab with hair regrowth: a case report. Clin Cosmet Investig Dermatol.

[14] Sukhatme S.V., Lenzy Y.M., Gottlieb A.B. (2008). Refractory dissecting cellulitis of the scalp treated with adalimumab. J Drugs Dermatol.

